# Screening based approach and dehydrogenation kinetics for MgH_2_: Guide to find suitable dopant using first-principles approach

**DOI:** 10.1038/s41598-017-15694-x

**Published:** 2017-11-14

**Authors:** E. Mathan Kumar, A. Rajkamal, Ranjit Thapa

**Affiliations:** SRM Research Institute & Department of Physics and Nanotechnology, SRM University, Kattankulathur, 603203 Tamil Nadu, India

## Abstract

First-principles based calculations are performed to investigate the dehydrogenation kinetics considering doping at various layers of MgH_2_ (110) surface. Doping at first and second layer of MgH_2_ (110) has a significant role in lowering the H_2_ desorption (from surface) barrier energy, whereas the doping at third layer has no impact on the barrier energy. Molecular dynamics calculations are also performed to check the bonding strength, clusterization, and system stability. We study in details about the influence of doping on dehydrogenation, considering the screening factors such as formation enthalpy, bulk modulus, and gravimetric density. Screening based approach assist in finding Al and Sc as the best possible dopant in lowering of desorption temperature, while preserving similar gravimetric density and Bulk modulus as of pure MgH_2_ system. The electron localization function plot and population analysis illustrate that the bond between Dopant-Hydrogen is mainly covalent, which weaken the Mg-Hydrogen bonds. Overall we observed that Al as dopant is suitable and surface doping can help in lowering the desorption temperature. So layer dependent doping studies can help to find the best possible reversible hydride based hydrogen storage materials.

## Introduction

Metal hydrides are the most technology relevant class of hydrogen storage materials employed to store hydrogen as a compact energy source as well as anode materials in the rechargeable batteries (Ex: commercial available nickel-metal hydride battery) for the portable applications^1,2^. It can be used in a wide range of other interesting applications such as aircraft fire detectors, hydrogen compression, and isotope separation^3–5^. Among these applications, storage of hydrogen in metal hydrides is most demanding to replace the fossil fuels, because of its clean combustion products and sustainability^6^. Solid metal hydride such as light element complex hydrides, Magnesium Hydride (MgH_2_) and alantes are the most promising materials with high hydrogen storage capacity. In particular, MgH_2_ has higher hydrogen content (7.6 wt %), highly abundant and low cost^7^. Moreover, the formation enthalpy of MgH_2_ (−76 kJ/mol) indicates it as a thermodynamically most stable system at ambient pressure and temperature which prevents the release of hydrogen from MgH_2_^8^. The desorption of hydrogen takes place around 300 °C at ambient pressure^9^. In addition, dehydrogenation kinetics studies are required to find the role of surface doping and underneath doping in the metal hydrides by considering layered structure (here, MgH_2_).

So far, quite a lot of research works are focused on destabilizing the metal hydride by either adding additives or substitutional doping leads to the reduction of H_2_ desorption temperature of the host material^10,11^. *Süleyman et al*. verifies that the reduction of desorption temperature is mainly due to the transformation from stable rutile structure to unstable fluorite structure of MgH_2_^12^. *Vajo et al*. reported that by adding of 0.5 equivalent concentration of MgH_2_ to LiBH_4_, the dehydrogenation enthalpy is lowered by 25 KJ/(mol of H_2_) compare to the pure LiBH_4_^13^. *Alapati et al*. reports, it is possible to screen the doping element, considering reaction enthalpy as screening parameter and it is found that additives like MgH_2_ can be used as a destabilizing agent for metal hydrides^14^. *Ahuja* and his co-workers furnish a mechanism for dehydrogenation in case of Nb doped MgH_2_ and also establish that vacancies don’t play a significant role in lowering the desorption temperature^15^. *Sun et al*. explains the importance of co-doping (Ni and Y) in MgH_2_. The Mg-H bond strength decreases mainly due to strong hybridization between dopant (Ni and Y) and the nearest H atoms^16^. *Li et al*. showed that Ti substitution at Al site is more favourable in sodium alanete (NaAlH_4_) and also it helps to reduce the desorption temperature^17^. *Morioka et al*. reported that potassium aluminium tetra hydrides (KAlH_4_) exhibits reversible decomposition under less pressure but the operating temperature is higher than the sodium alanate^18^. The 6.25% doping of Al, Ni, Ti, V and Fe in the Mg_16_H_32_ system shows less desorption temperature, faster diffusion time and less stability than pure Mg_16_H_32_^19^. The mechanical milling was used for making the MgH_2_-Transition metal nanocomposite powders by *Liang et al*. The formation enthalpy and entropy of the host system were not changed by mechanical milling with transition metals but the desorption energy was reduced drastically for Ti and V cases^20^. *Khatabi et*. *al*. considers 3d and 4d elements (Sc, Ti, V, Cr, Y, Zr, Nb and Mo) as dopants to reduce the desorption temperature of MgH_2_. The bond weakening was observed in all the cases which help to confirm the destabilization in the doped system^21^.

Dehydrogenation mechanism on MgH_2_ layers is studied by various research groups. Mostly, the transition metals dopants are used to improve the kinetics of MgH_2_. Experimentally, it is achievable to reduce the absorption/desorption kinetics of H_2_ by mechanical milling of MgH_2_ and adding transition metals^22,23^. The pathway for re-combinative desorption of one in-plane and one bridging H atom from the MgH_2_ (110) surface is found to be the lowest energy barrier as reported by *Du et al*.^24^ and this argument is in good agreement with the experimental result. *Wang et*. *al* reported that the kinetics can be improved by Cu dopant in the MgH_2_ (110) plane and activation energy is reduced by the formation of cluster (CuH_4_) near the vicinity of dopant^25^. *Dai et*. *al* notices that transition metal doping results in weakening of Mg-H bond and there is a maximum chance to form the thermodynamically unfavorable intermediate phase^26^. *Wu et al*. reported that for (110) surface of MgH_2_, the H_2_ desorption barrier energy is lower compared to the MgH_2_(001) surface^27^. *Sun et al*. proves that combination effect of strain and doping reduces the barrier energy for dehydrogenation of MgH_2_ (110) surface^28^. The catalytic effect of Ti is explained by *Wang et al*., i.e Ti doped MgH_2_ (110) surface, the barrier energy of H_2_ desorption is reduced by 0.41 eV compares to the pure case^29^. In all these studies, destabilizing metal hydrides is the major task performed, but their handling tools were individual and different. Our focus is not only to destabilize the material but also to maintain the stability of material to some extent which can aid in easily adsorbing and desorbing the hydrogen in ambient conditions. Therefore, we prefer a combo of study that can precisely scan for the proper dopant as per our demand (retaining some property like same bulk modulus value and more than 7% gravimetric density).

In this work, we carry out density functional theory (DFT) based calculations to examine the thermodynamics and kinetics of the bulk and (110) surface of MgH_2_ system by inserting three different types of dopants; neutral, n-type and transition metal. Consequently, in this work we propose screening based study (the parameters such as desorption temperature, gravimetric density and bulk modulus) to select an appropriate dopant for reducing desorption temperature of MgH_2_. For n-type and transition metal doped systems, the change in the electronic structure of MgH_2_ is observed which clearly emphasize its structural in-stability. Electron localization function (ELF) calculation and charge population analysis are performed to reveal the dopants effects on bonding properties of MgH_2_. cNEB calculation is used to obtain H_2_ desorption energy barriers for pure and doped MgH_2_ (110) surface. The layer dependent doping effect on the dehydrogenation kinetics is studied in detail for all the chosen systems. Molecular Dynamics (MD) study is done for pure and Al doped MgH_2_ system and discussed in the last section.

## Result and Discussion

### Structure Information

**Figure 1:**
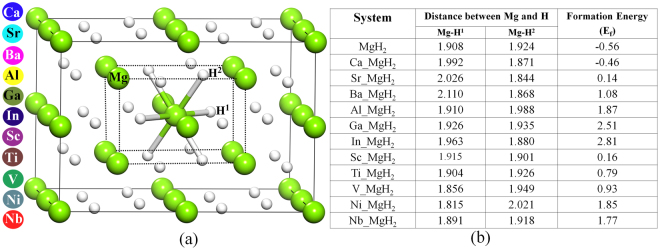
(**a**) Demonstrate the (2 × 2 × 2) supercell of MgH_2_. The smaller dotted cell represents the unit cell of MgH_2_. Mg and hydrogen atoms are denoted with green and white sphere. Whereas the H^1^ and H^2^ type hydrogen atoms are clearly shown in the unit cell. The dopant element consider in this work are shown in the left panel by various colour dots. (**b**) Table as figure represents the distance between Mg/dopant and H^1^ and H^2^ atoms, formation energy (E_f_).

In our calculations, we consider a rutile-type Magnesium hydride (α-MgH_2_) of tetragonal symmetry (P42/mnm) with initial lattice parameters of a = b = 4.501 Å and c = 3.010 Å. We identify two different types of hydrogen atoms namely, H^1^ and H^2^ (present in MgH_2_ crystal) based on atomic coordinates of Mg and H atoms. In this structure, each Mg atom is octahedrally coordinated by six H atoms, where two H atoms (labelled as H^1^) lie along [110] direction and four H atoms (labelled as H^2^) lie on the (110) plane and each H atom is coordinated by three Mg atoms (see Fig. 1a). The calculated (optimized) lattice parameters for bulk MgH_2_ are a = b = 4.435 Å and c = 2.962 Å and the bond length of Mg-H^1^ and Mg-H^2^ is 1.908 Å and 1.924 Å respectively, which agree well with the previously reported results^30^. Here we consider three different categories of dopants to study the effect of doping on hydrogen desorption temperature, bulk modulus and gravimetric density of MgH_2_. The categories of dopants are; (i) neutral dopant (Ba, Ca, Sr), (ii) n-type dopant (non-transition metal, Al, Ga, In) and (iii) transition metal (TM) atom as the dopant (Sc, Ti, V, Ni, Nb). To deal with low doping concentration, supercell approach is essential and hence we took a (2 × 2 × 2) supercell of MgH_2_ as the reference system. The bond length change for all the doped MgH_2_ is shown in Fig. 1b along with the formation energy value. The formation energy (E_f_) can be written as

1 $${{\rm{E}}}_{{\rm{f}}}={E(\mathrm{Mg}}_{16-{\rm{n}}}{{\rm{D}}}_{{\rm{n}}}{{\rm{H}}}_{32})-{E(\mathrm{Mg}}_{16}{{\rm{H}}}_{32})-{\rm{E}}({{\rm{D}}}_{{\rm{n}}})+{E(\mathrm{Mg}}_{{\rm{n}}})$$

**Figure 2:**
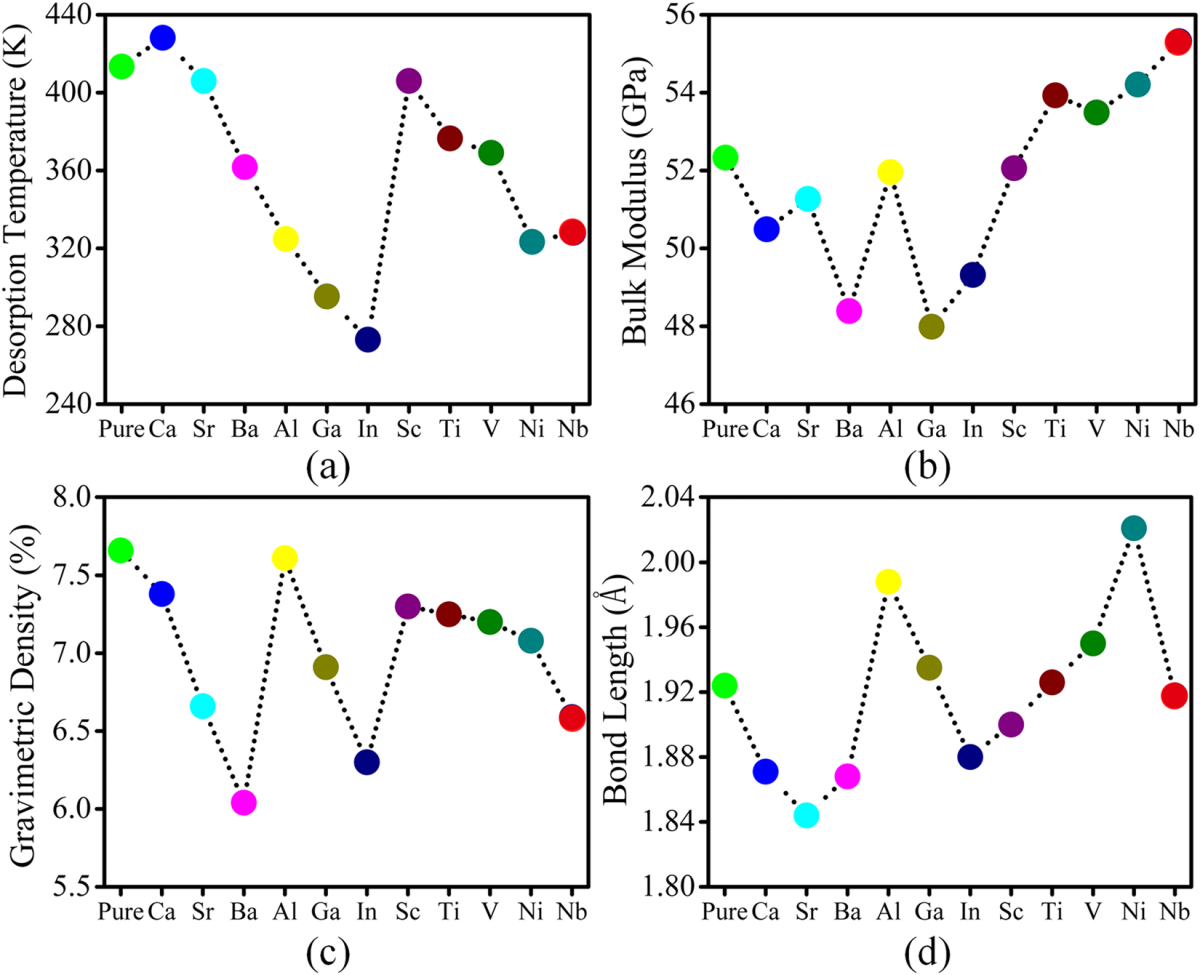
Calculated (**a**) desorption temperature, (**b**) bulk modulus, (**c**) gravimetric density and (**d**) Bond length (Mg-H^1^) variation for pure and doped MgH_2_ systems. The colour dots are for same dopant as consider in Fig. 1. The dotted lines are only for guide to the eye.

Where E(Mg_16-n_M_n_H_32_), E(Mg_16_H_32_), E(D_n_) and E(Mg_n_) are the total energy of doped MgH_2_, pure MgH_2_, bulk form of dopant and bulk Mg respectively. Here n is the number of dopant atom added and the number of Mg atom removed from the host system. For all the dopant configurations, the value n in the subscript is one. Firstly, we performed the preliminary analysis by considering the parameters such as hydrogen desorption temperature, bulk modulus, change in the bond length and gravimetric density to screen the best possible dopant to tune MgH_2_ as a potential candidate for hydrogen storage material, as shown in Fig. 2.

### Screening Factors: Formation Enthaply, Bulk Modulus and Gravimetric Density

Destabilizing MgH_2_ by doping is the most versatile and useful technique adopted by the researchers. But the random doping will not help in finding the explicit solution. Therefore, for the necessary standardization, we need a combo of these preliminary studies as “screening based analysis”. This analysis will serve as a common tool to determine the structural stability for any metal hydrides. Primitively, to identify the novel and effective dopants, comparison of formation enthalpy is needed to check the stability of doped MgH_2_ with its pure crystal. The formation enthalpy at 0 K is calculated by using the following equation,

2 $$\begin{array}{c}{\rm{\Delta }}\end{array}{{\rm{H}}(\mathrm{Mg}}_{16-{\rm{n}}}{{\rm{D}}}_{{\rm{n}}}{{\rm{H}}}_{32})=1/16[{{\rm{E}}}_{{\rm{tot}}}({{\rm{Mg}}}_{16-{\rm{n}}}{{\rm{D}}}_{{\rm{n}}}{{\rm{H}}}_{32})-(16-{\rm{n}}){\rm{E}}({\rm{Mg}})-{\rm{nE}}({\rm{D}})-16{\rm{E}}({{\rm{H}}}_{2})]$$

Where E_tot_(Mg_16-n_D_n_H_32_), E(Mg), E(D) and E(H_2_) are the total energy of doped MgH_2_, bulk Mg, bulk of dopant and H_2_ molecule respectively. To find the formation enthalpy of pure MgH_2_, the value of n should be zero. The formation enthalpy of MgH_2_ is −0.56 eV and the value is well matched with the previously reported theoretical work^31^. It is to be noted that the high negative value of formation enthalpy leads to high desorption temperature and sluggishness of hydrogen desorption kinetics for MgH_2_.

The formation enthalpy values for all class of doped system is negatively small compared to the pure MgH_2_ except Ca doped system and it assures that the destabilization happens in all the other doped MgH_2_. From Fig. S1 (see supplementary information), the formation enthalpy values for neutral type dopants are in the range of −0.48 eV to −0.58 eV and this result revealed the less structural change and its comparable stability with the pure MgH_2_. For n-type doped system, the formation enthalpy values (−0.37 eV to −0.44 eV) are negatively smaller than pure and other class of doped MgH_2_ systems. The range of formation enthalpy for transition metal doped case is about −0.44 eV to −0.55 eV in which Ni and Nb doping lead to lower formation enthalpy compared to other transition metal dopants. From this analysis, except Ca dopant all other dopants are found to be favorable agents for destabilizing the MgH_2_ system. Furthermore, it can be inferred that the study of formation enthalpy alone cannot determine the best possible dopant to make MgH_2_ as reversible hydrogen storage materials.

The desorption temperature is directly proportional to formation enthalpy as per Gibbs free energy relation and can be estimated by the following equation,

3 $${\rm{T}}={\rm{\Delta }}{\rm{H}}/{\rm{\Delta }}{\rm{S}}$$

Where ∆S is the dehydrogenation reaction entropy that can be approximated as *∆S*(*H*_2_) = *130*.*7* *J/molK* ^14^. From Figs 2a and S1, we disclose that the desorption temperature decreases with decrease in formation enthalpy. This direct relation has followed the same trend as formation enthalpy value.

Secondly, here we estimate and discuss the Bulk modulus (B_o_) of each system, which is considered as another screening factor depending on the pressure, to assess the stability of pure and doped MgH_2_ system. The bulk modulus is determined by using of Birch-Murnaghan equation via fitting of total energies with the cell volume of the system (see Fig. S2)^32^. To avoid the pulay stress problem, all the geometries are relaxed with energy cut-off of 450 eV. The bulk modulus of all systems is estimated and is shown in Fig. 2b. The volume change (compressibility) in the system is indirectly proportional to the bulk modulus of the system. Therefore, the system with low bulk modulus has high compressibility (volume change), which in turn reduces the system stability. The bulk modulus for the pure case is found to be about 52.33 GPa and it is well matched with the previously reported theoretical work^32^. The change in volume of the systems has been estimated for 100 atm applied pressure and it is demonstrated in Table 1 (to be noted that operating applied pressure is 1 to 100 atm as per DOE target). At this condition, the change in volume for pure system is about 0.0193%. For neutral doped case, the bulk modulus lies in the range of 48 GPa to 51 GPa and for Sr doped case the value is 51.26 GPa, which is close to the pure case. The change in volume for the neutral doped system is increased to 0.0209% at 100 atm applied pressure and this leads to destabilization of MgH_2_. For n-type doped system the bulk modulus value is in the range of 48 GPa to 52 GPa. For Al doped system, the change in volume is 0.0194% for 100 atm applied pressure and is almost equal as that of pure case, this indicates that it can be a better system with retaining property (retaining property). The higher bulk modulus is found for transition metal doped MgH_2_ and it is in the range of about 52 GPa to 55 GPa. Moreover, the Sc doped MgH_2_ has the similar change in volume 0.0194% for applied pressure as of pure MgH_2_ and it is higher than other transition metal doped MgH_2_. It is to be noted that mostly all transition metal dopant increases the stability of the MgH_2_ system by considering applied pressure as dehydrogenation parameter. All other dopant decreases the stability of the system. The observation here is to relate the stability of the system with the applied pressure. The effect of changes in the volume is looking very small (0.0183 to 0.0209%), since we consider 100 atm as an externally applied pressure. We also consider the external pressure of 10 GPa and estimated the total volume change (about 18% volume change is observed) for pure and doped systems (the values are given in the Table S1). To be specific Al and Sc doped system acquires much needed stability to make a recycled reaction and less alternation in structure compared to other doped systems. In comparison with desorption temperature analysis, we shorten the list of dopants by bulk modulus values but still, it is not in the situation to fix the proper dopants to dehydrogenate the bulk MgH_2_ at the lower temperature and moderate pressure.

**Table 1:** The bulk modulus value and corresponding percentage in volume change for pure and doped MgH_2_ systems at 100 atm applied pressure

System	Bulk modulus (GPa)	% of ΔV (at 100 atm Pressure)
MgH_2_	52.33	0.0193
Ca_MgH_2_	50.49	0.0201
Sr_MgH_2_	51.27	0.0195
Ba_MgH_2_	48.40	0.0209
Al_MgH_2_	51.96	0.0194
Ga_MgH_2_	47.99	0.0211
In_MgH_2_	49.32	0.0205
Sc_MgH_2_	52.06	0.0194
Ti_MgH_2_	53.92	0.0187
V_MgH_2_	53.49	0.0189
Ni_MgH_2_	54.21	0.0186
Nb_MgH_2_	55.32	0.0183

Thirdly, we calculated the gravimetric density of pure and doped systems (shown in Fig. 2c) which can be considered as the important screening factor. For pure MgH_2_ the gravimetric density is found to be about 7.6%. The gravimetric density should be in the range of 7% (DOE target) for the doped MgH_2_ system, so that it can be used for the commercial purposes. We found that for Ca, Al, Ga, Sc, Ni, Ti and V doped MgH_2_ system, the gravimetric density value is about 7% (see Fig. 2c) and for other doped (Ba, Sr, In and Nb) system the gravimetric density value is lower than 7%. But as discussed in previous sections, by considering the desorption temperature and bulk modulus as screening factor we concluded that the dopants like Ca, Ba, Ga, In, Ti, V, Ni and Nb are not suitable for tuning MgH_2_ as better reversible hydrogen storage material. Consequently, As per our screening based approach, we found that Al and Sc doped MgH_2_ systems are better hydrogen storage materials when compared to pure and other doped systems.

To affirm above argument, the average bond length of hydrogen atom with its nearest host atom (Mg) is measured in all the doped system and it is compared with the pure MgH_2_ (see Fig. 2d). As we mentioned earlier, the bond length between Mg and H^2^ atom is about 1.924 Å for the pure case. The average bond length between Mg and H^2^ is higher in Al, Ga, Ti, V and Ni doped systems and the values are 1.988 Å, 1.935 Å, 1.926 Å, 1.95 Å and 2.021 Å respectively. These results illustrate that the formation of hydrogen cluster in the vicinity of dopants is possible for Al, Ga, Ti, V and Ni doped MgH_2_. It is to be noted that in Ga, Ti and V doped cases the average bond length between Mg and H^2^ is less compared to Al and Ni doped MgH_2_ system. Overall we observed that Al doped system can be the best dopant to tune MgH_2_ as the Hydrogen storage material.

Considering all the screening factors (gravimetric density, formation enthalpy and bulk modulus), we recognize that n-type dopant (Al) and transition metal dopant (Sc) are the superior agents to destabilize the MgH_2_ along with retaining the host property. Furthermore, the neutral series elements are not at all found to be suitable. Thus, these combined preliminary studies are suggested to be a good choice (as per our results) to determine the suitable dopant to destabilize the metal hydrides. The random doping in the field of destabilizing the metal hydrides can be replaced by our choice of study which will effectively reduce the cost and time for experimentalists in this field.

### Electronic Structure

Further, we figured out doping effects in MgH_2_ by comparing the electronic structure of both pure and doped MgH_2_ systems. From total density of states (TDOS) plot, it is observed that pure MgH_2_ has 3.24 eV band gap by using PBE-PAW method and it matches well with previously reported work^31^. There is an absence of magnetism because of its flawless symmetry of spin up and down states. The valence band is mostly occupied by the combination of s, p states of Mg atom and s state of H atom. The conduction band is dominated by s states of Mg atom (see Fig. S3a). In the case of Ba, Ca and Sr (neutral atom) doped MgH_2_ systems, almost similar electronic structure and band gap (around 3 eV) are observed when compared to the pure MgH_2_ and are depicted in Fig. S4. The absence of defect states near the Fermi energy substantiates the less influence of neutral dopants on destabilization of MgH_2_.

**Figure 3:**
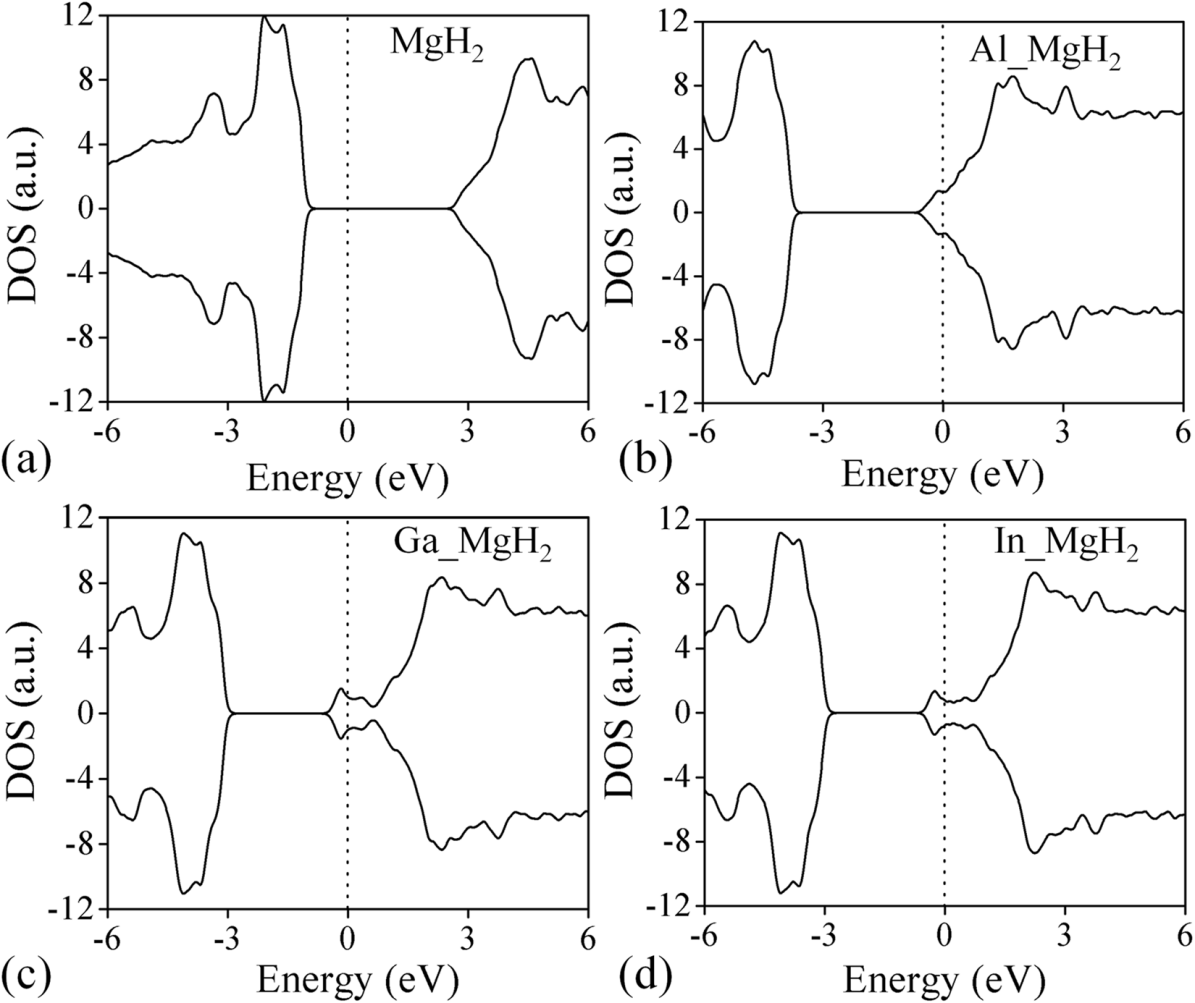
Total density of states (**a**) pure MgH_2_, (**b**) Al_MgH_2_, (**c**) Ga_MgH_2_ and (**d**) In_MgH_2_. The vertical dotted line represents the position of the Fermi level.

Figure 3 shows TDOS of n-type doped (Al, Ga and In) MgH_2_ system. In all the cases, the Fermi level shifts towards the conduction band due to doping of one-electron per supercell as contributed by the dopants. The donor states at the Fermi level is mainly occupied by s state of H, s states of Mg and p state of dopant, which is clearly demonstrated in Fig. S3b. It is known that higher the density of occupied state near Fermi level, the system becomes less stable. Consequently, the localized states lower the stability of MgH_2_ and cause the negative lowering of formation enthalpy which directly impacts on dehydrogenation temperature. We performed the TDOS for pure and Al doped MgH_2_ using HSE06 calculation for confirming the accuracy of calculation. From the TDOS plot, it is observed that pure MgH_2_ has 3.42 eV band gap estimated using HSE06 method and we found that almost similar electronic structure is observed for pure and Al doped MgH_2_ cases when compared to the PAW-PBE method (see Fig. S5).

The band gap and stability of the MgH_2_ system are altered by doping of transition metal atoms. Transition metals doped MgH_2_ is magnetic in nature as confirmed from the TDOS (see Fig. S6a–f). In the case of pure MgH_2_, an interaction between Mg and H is electrostatic in nature^31,33^. Whereas in the transition metal doped MgH_2_ case, H atoms get closer to the dopant due to stronger metal-hydrogen bond and hence cluster is formed in the vicinity of dopant (confirmed by bond length analysis). The PDOS of transition metal atom’s d orbitals is shown in Fig. S7. The three main peaks below Fermi level are locally arranged by the metal-ligand interaction, which can be explained by crystal field theory. As per the crystal arrangement of MgH_2_, a central metal atom is surrounded by six hydrogen atoms (slightly deformed octahedral positions). The different orientation in d orbital $({d}_{{z}^{2}},\,{d}_{{x}^{2}-{y}^{2}},\,{d}_{xy},\,{d}_{xz},{d}_{yz})$ gets affected by surrounding atoms positions and bring about the change in electronic arrangement and thermodynamic properties. In octahedral crystal field, five degenerate d orbitals split into two groups with different energy levels namely e_g_ $({d}_{{z}^{2}},\,{d}_{{x}^{2}-{y}^{2}})$ and t_2g_ $({d}_{xy},\,{d}_{xz},\,{d}_{yz})$. Furthermore, the e_g_ orbital is affected only by surrounding H atoms (filled mainly by bonding and anti-bonding orbitals) and the t_2g_ orbital is unaffected. The occupied states near the Fermi level is largely dominated by the hybridized state of d orbital of transition metals and its weak H atom (see Fig. S8). In comparison, with all transition metal (Sc, Ti, V, Ni, Nb) doped system; Sc doped MgH_2_ shows high electron density at the Fermi level which reflects the instability of the system. The probability of electron density at the Fermi level decides the system instability and vice-versa.

### Structural bonding Analysis

**Figure 4:**
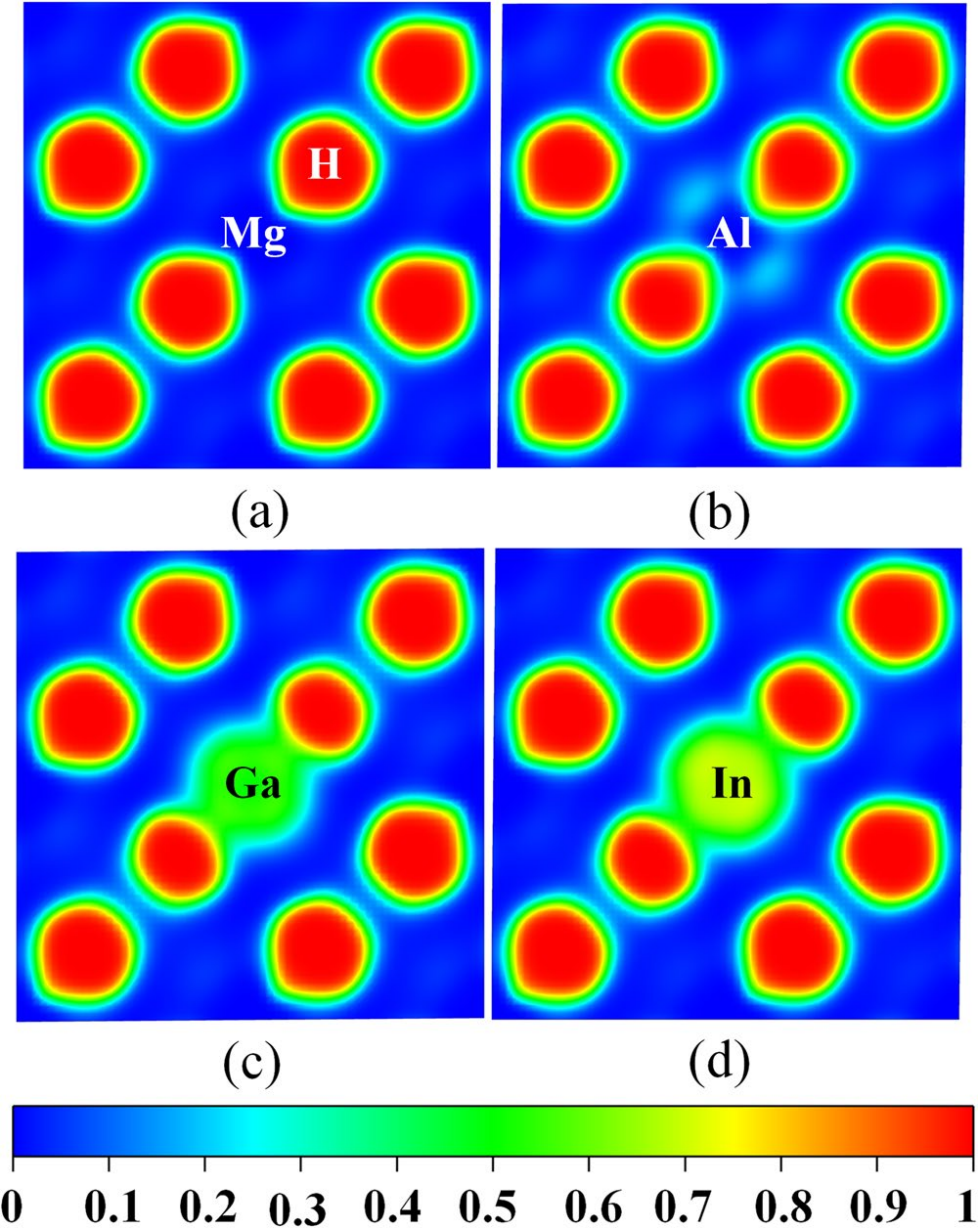
The 2D plot of Electron Localization Function for (**a**) pure MgH_2_, (**b**) Al_MgH_2_, (**c**) Ga_MgH_2_ and (**d**) In_MgH_2_. The red and blue region indicates high and low localization also can be estimate from the bar scale.

To examine bonding nature of pure and doped MgH_2_ systems, we analyse the electron localization function. The ionic interaction between Mg and H atom is clearly illustrated by the ELF plot in Fig. 4a, which is in well-agreement with a reported work^34^. Hydrogen atoms are surrounded by high localization (indicated by red contours) of value around 0.98 and less localization (indicated by blue contours) are observed around Mg atoms. For a neutral doped system, change in electron localization at dopant site and reduction in the spherical shape of H atoms (high localization area) is observed. This prediction demonstrates that the formation of covalent bond is more favourable than the ionic bond in neighbourhood of the dopant site (see Fig. S9a–d). In Figs 4 and S10, the same trend has been observed for n-type and transition metal doped MgH_2_ system. High localization around dopant region and reduction in bond length between the transition metal and its nearer H atoms leads to cluster formation. To clarify the above analysis, bond population between the dopant-H^1^ (D-H^1^) and Mg-H^1^ is calculated and it is listed in Table 2. The bond population is calculated by considering the linear combination of atomic orbitals (LCAO) basis set, which estimates the localization of electrons in the system, as available in CASTEP code^35^. The observation of neutral dopants by ELF analysis is rechecked by charge population analysis. The Ca and Sr doped MgH_2_ has less positive value which indicates the less covalency than other categories of dopants. The Ba doped MgH_2_ has same charge population value (−0.47) like pure MgH_2_ which retain its ionic character in the bonding. The highest charge population value for D-H^1^ (0.66) has occurred for Al doped MgH_2_ system. This case indicates that the formation of the cluster with higher stability reduces the bond stability of Mg-H^1^. It is to be noted that the charge population value is positive and higher than 0.50 for n-type and transition metal doped MgH_2_ system, except for Ga doped system. The charge population for Mg-H bond varies in-between −0.03 to −0.17 for all the doped system which is quite low when compared to the pure MgH_2_ system. This result proves that destabilization occurred in doped MgH_2_ by the reduction in ionicity on the neighbourhood of dopant region. Hence, the bonding nature of doped MgH_2_ system shows mainly ionic but as the D-H^1^ bond is covalent, (n-type and transition metal doped MgH_2_) it causes destabilization of Mg-H bond in the MgH_2_ systems.

**Table 2:** Bond length and charge population between Mg and H^1^ atom, dopant (denoted as D) and H^1^ atom in pure and doped MgH_2_ systems.

System	D-H^1^ bond		Mg-H^1^
	Bond length (Å)	Charge population	Charge population
MgH_2_	—	—	−0.48
Ca_MgH_2_	2.11	0.17	−0.07
Sr_MgH_2_	2.23	0.18	−0.16
Ba_MgH_2_	2.41	−0.47	−0.41
Al_MgH_2_	1.79	0.66	−0.07
Ga_MgH_2_	1.86	0.33	−0.04
In_MgH_2_	2.03	0.55	−0.12
Sc_MgH_2_	1.99	0.56	−0.05
Ti_MgH_2_	1.87	0.51	−0.17
V_MgH_2_	1.80	0.52	−0.14
Ni_MgH_2_	1.68	0.55	−0.03
Nb_MgH_2_	1.90	0.58	−0.10

### Dehydrogenation Kinetics

Even as we have studied in detail about few screening factors to find the best possible dopant, here we study how the doping position at various layers affect the hydrogen desorption kinetics for the pure and doped MgH_2_ (110) surface. To find out the energy barrier for H_2_ desorption, cNEB calculations are performed for pure and doped MgH_2_ (110) surfaces. In the model, we used three layers of (2 × 2 × 1) supercell of MgH_2_ (110) surface, which consist of 72 atoms and 15 Å vacuum along z direction is taken to avoid the spurious interaction. We calculate the value of total energy (in eV) of Al doped at second and third layer of MgH_2_(110) surface (considering three layer system) with reference to the total energy of Al doped at first layer of MgH_2_(110) surface (see Table S2). The total energy of Al doped at first layer is taken as 0 eV. From Table S2, the Al doping in second and third layer needs 0.58 eV and 0.64 eV compare to the first layer. Overall we can infer that surface doping is easier compare to underneath doping.

**Figure 5:**
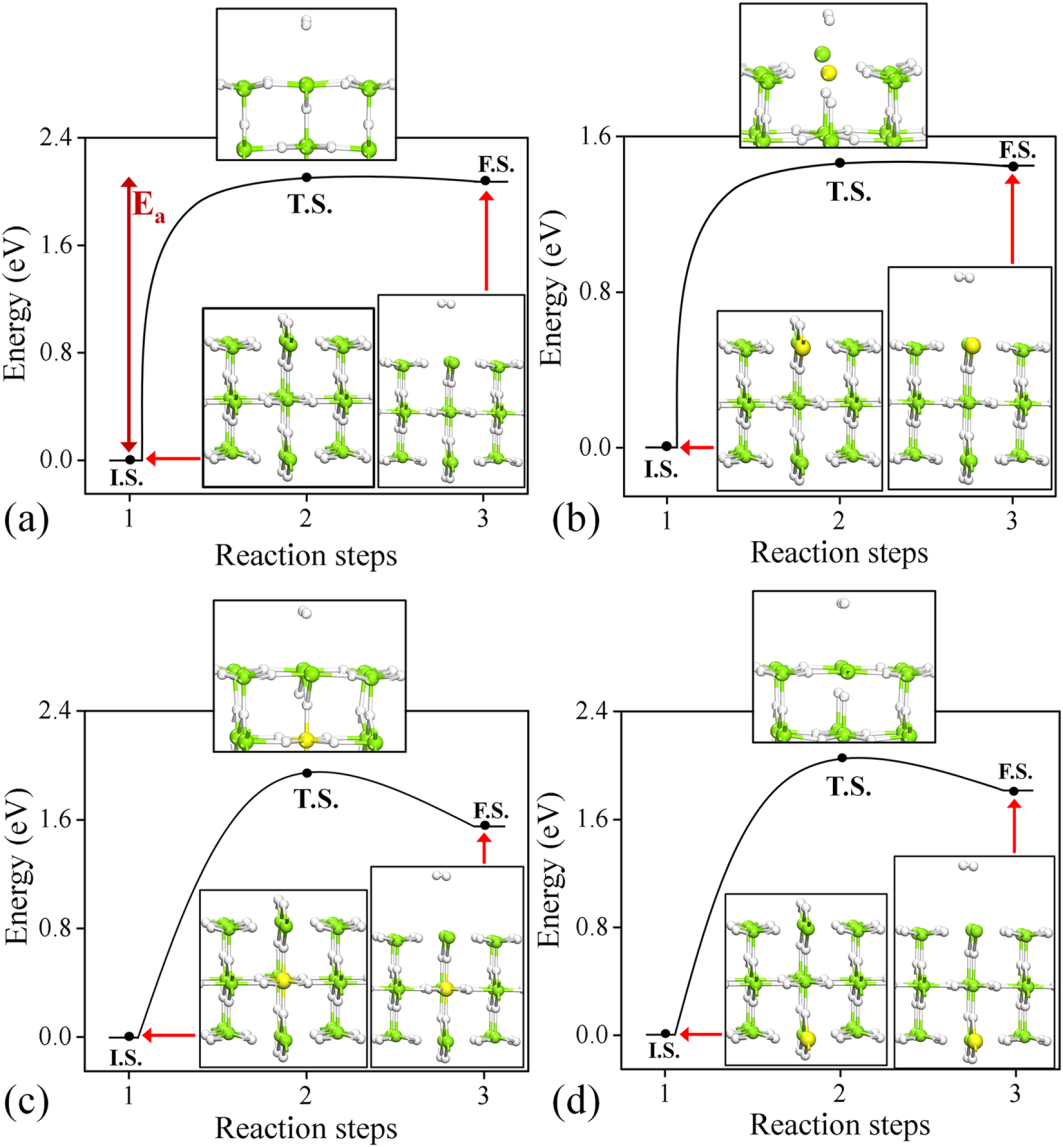
The barrier energy curve for the desorption of hydrogen molecule from top layer of (**a**) pure MgH_2_, (**b**) Al doped at top layer of MgH_2_ (110) structure, (**c**) Al doped at second layer of MgH_2_ (110) and (**d**) Al doped at third layer of MgH_2_ (110) structure. The inset figures are the relaxed structure corresponds to initial state (I.S.), Transition state (T.S.) and final state (F.S.). The black line connecting I.S., T.S. and F.S. are only for guide to the eye. E_a_ denote the activation barrier energy.

**Figure 6:**
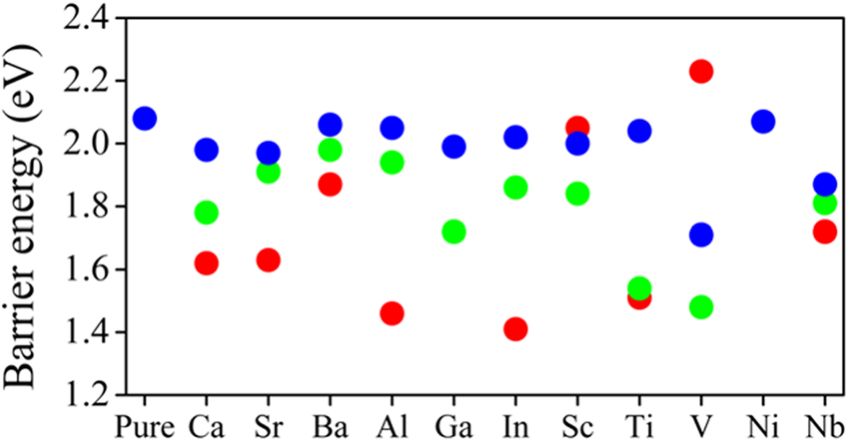
Barrier energy of H_2_ desorption from the top layer to far position considering the doping at various layer of MgH_2_ (110) structure and for all doped system. Red, green and blue colour solid circle represents barrier energy value for H_2_ desorption in case of doping site at first, second and third layer of pure and doped MgH_2_ (110) surface.

The initial states (IS) are chosen as the relaxed equilibrium configurations of pure and doped MgH_2_ (110) surface. The final states (FS) are made by removing a pair of H atoms from the top layer and reintroducing as an H_2_ molecule at a distance of about 4.0 Å from the surface. The hydrogen desorption barrier energy for pure case (see Fig. 5a) is found to be about 2.08 eV. Here we estimate the H_2_ desorption barrier energy considering all type of doping in first, second and third layer of MgH_2_ (110) surface, as shown in Fig. 6. The barrier energy in case of Al doped (at first layer) MgH_2_ (110) surface is 1.45 eV (shown in Fig. 5b) and it is about 0.6 eV lower than the pure case. Whereas for second and third layer Al doped MgH_2_ (110) system, the barrier energy is 1.94 eV and 2.05 eV respectively (shown in Fig. 5c and d). More specifically, for Al doped (at third layer) MgH_2_ (110) system the H_2_ desorption energy is similar to the pure system, indicating a lower impact of deeper doping. It is to be noted that, the shallow doping has the key role in tuning desorption barrier energy. At transition state, the H-H bond length is about 1.3 Å and it is almost similar for pure and underneath doping configurations. In case of first layer (Al atom) doped MgH_2_, the H-H bond length is around 0.82 Å at transition state. The results indicate easy formation of H_2_ molecule in case of first layer (Al atom) doped MgH_2_. We have done the similar calculation steps considering all other dopants and we found that Ca, Sr, Ba, In, Ti and Nb doped cases follows the same trend like that of Al doped MgH_2_ (110) surface (see Fig. 6). Interestingly, in the case of Sc and V doped MgH_2_ (110) system, the H_2_ desorption energy for first layer doped system is higher than the second and third layer doped system (see Fig. 6). In the case of Ni and Ga doped MgH_2_ (110) system, the final geometry for the first layer doped case is more distorted which resist to finding the barrier energy for molecular hydrogen desorption. The cluster formation and charge population (D-H^1^ bond) for bulk Al doped MgH_2_ play the same role for reducing desorption energy barrier in the first layer doped MgH_2_ system. Overall we found that the lowest desorption barrier energies are 1.41 and 1.46 eV for In and Al doped MgH_2_ (110) surfaces. Hence, apart from the screening based approach to find suitable dopants, we have also proposed in detail that the layer dependent doping can also change the desorption temperature of H_2_. Moreover, comparing surface doping and underneath doping, it is found that surface doping is preferable because it effectively reduces the hydrogen desorption energy.

### Radial Distribution Function

**Figure 7:**
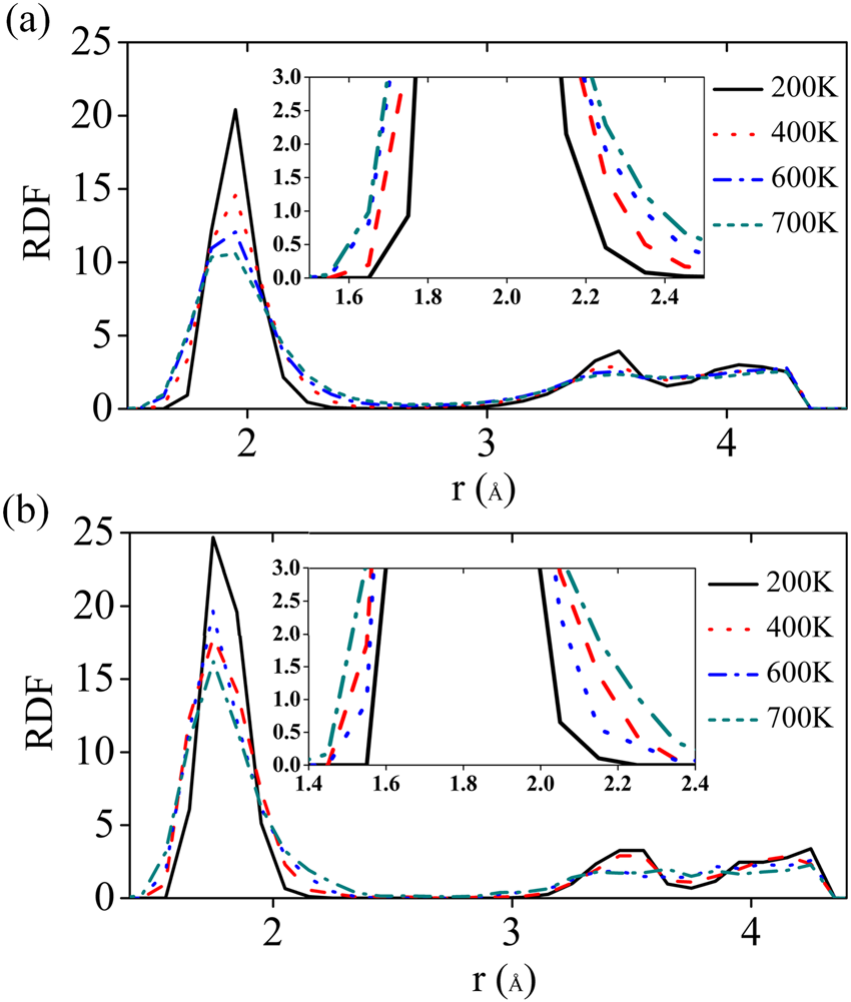
The variation of Radial distribution function as a function of distance r (Å) from the reference atom of (**a**) pure and (**b**) Al doped MgH_2_(110) surface for four different temperatures i.e. 200 K, 400 K, 600 K and 700 K are shown by black, blue, red and magenta coloured lines respectively. Insert shows the RDF considering the finer window of r(Å), showing the broadening of peak with increase of temperature.

The stability and structural changes of pure and Al doped (surface) MgH_2_(110) surfaces are also validated by ab-initio molecular dynamics (MD) simulation. We plotted the radial distribution function (RDF) as a function of distances (r) for various applied temperatures 200 K, 400 K, 600 K and 700 K and it is shown in Fig. 7a and Fig. 7b. Here the RDF indicates the density of nearest atoms from a reference atom. The first sharp peak indicates the nearest neighbor atom distances from the reference atom. Considering Mg as reference atom for the pure case, we found that the nearest Mg-H bond distance is around 1.9 Å and for Al doped case the Al-H bond distance is about 1.7 Å considering Al as reference atom (see Fig. 7). The reduction in the Al-H bond length for Al doped MgH_2_ case proves the clusterization of hydrogen atoms in the vicinity of dopant site. The reduction in the peak height and broadening of the peak with increase in temperature clearly explain the destabilization of pure and Al doped MgH_2_ (110) surface and it is shown in the insert of Fig. 7. The total radial distribution function is calculated by integrating the area for all the temperatures and it is confirmed that the same number of hydrogen atoms is surrounding the reference atom for all cases (see Fig. S11). Hence, the octahedral complex is stable in higher temperature region for both pure and Al doped MgH_2_(110) surfaces. So we again confirm that the formation of most stable cluster reduces the bond stability of Mg-H and temperature plays a major role in further destabilization of the system.

## Conclusions

First-principles based calculations were performed to study the effect of (i) various type of doping and (ii) layer dependent doping, on the dehydrogenation of MgH_2_. By calculating the barrier energy during H_2_ desorption from pure and doped MgH_2_ (110) surface, we observed that the doping sites based on layers have a significant role. If the doping is on the first or second layer, the barrier energy is essentially reduced, whereas doping at the third layer does not affect the dehydrogenation kinetics. The barrier energy for Al doped (in the first layer) MgH_2_ (110) surface is found to be 1.45 eV, which is 0.6 eV lower than the pure case. Hence the doping type and site can change the dehydrogenation kinetics of hydride materials. The formation of the cluster, reduction in the bond-stability of Mg-H and destabilization of the system is confirmed via molecular dynamics approach. Furthermore, the screening based approach helps to conclude that Al and Sc are the suitable dopants, which can destabilize the MgH_2_ via maintaining the gravimetric density and bulk modulus. In the case of Al and Sc doped systems, desorption temperature is about 325 K and 405 K respectively and it is lower than the pure case (413 K). The gravimetric density is 7.61% and 7.3% in case of Al and Sc doped MgH_2_ system respectively. The bulk modulus is found to be about 51.96 GPa and 52.06 GPa for Al and Sc doped MgH_2_ system, which is similar to pure MgH_2_ system (52.33 GPa). Particularly in Al doped MgH_2_ case, the charge population for D-H^1^ bond is 0.66 which is larger than other doped MgH_2_ system. The positive values (D-H^1^ bond) of all other doped system illustrates that Mg-H bonds are unstable when compared to the pure MgH_2_ system. Overall, we conclude that this approach can also be used to find the suitable dopants for other hydride materials and the effect of layer dependent doping can be primarily observed.

### Computational Details

We used spin polarized density functional theory as implemented in the Vienna ab-initio simulation package (VASP)^36^. The generalized gradient approximation (GGA) was introduced for the exchange and correlation effects at Perdew Burke Ernzerhof (PBE)^37^ and the potentials of the atoms were specified by the projected augmented wave (PAW)^38^ method. The energy cut-off is taken equal to 450 eV and the Brillouin zone integration within the Monkhorst Pack scheme with 5 × 5 × 7 k-point mesh in the reciprocal space is considered. For long-range Van der Waals interaction the Grimme’s method (DFT-D2) was used with PBE functional (denoted as PBE + D)^39^. All the structures were optimized until the total energy converged to less than 10^−5^ eV per atom and the maximum force converged to lower than 0.001 eVÅ^−1^. The barrier energy calculations were done by climbing image nudged elastic band (cNEB) method^40^. The climbing image is a small modification to the NEB method in which the highest energy image is driven up to the saddle point. The cNEB calculation was carried out by four intermediate images that connected between the initial and final states. For bulk based calculations we took (2 × 2 × 2) supercell and for surface calculations (2 × 2 × 1) supercell of MgH_2_ (110) plane with 15 Å vacuum along c-axis as the model geometry was used. For the bulk case, the amounts of doping considered here are 6.25% and for surface, the doping percentage is 4.17%. To check the stability of MgH_2_ (110) surface, we have used ab-initio molecular dynamics simulations. The NVT ensemble was used for varying the temperature range like 200 K, 400 K, 600 K and 700 K considering Nose thermostat^41^. For the equilibration, we used one thousand time steps with each step of 1fs long.

## Electronic supplementary material

Supplementary Information
