# Supplementary material for: Screening based approach and dehydrogenation kinetics for MgH2: Guide to find suitable dopant using first-principles approach
Source: Sci Rep. 2017 Nov 14;7:15550. doi: 10.1038/s41598-017-15694-x (PMC5686123; doi:10.1038/s41598-017-15694-x)
Supplement: Supplementary file 1 — Supplementary Information [file 41598_2017_15694_MOESM1_ESM.pdf]

Supplementary Materials

Screening based approach and dehydrogenation kinetics for MgH_2: Guide to find suitable dopant using first-principles approach

E. Mathan Kumar, A. Rajkamal and Ranjit Thapa*

SRM Research Institute & Department of Physics and Nanotechnology, SRM University, Kattankulathur 603203, Tamil Nadu, India

*Corresponding Author E-mail: ranjit.t@res.srmuniv.ac.in, ranjit.phy@gmail.com; Tel. No.: +91-44-27417918, Fax: +91-44-27456702

Table S1: Volume change in percentage for pure and doped MgH_2 at 10 GPa applied pressure

Table S2: Total energy difference for the three layer doping configuration (Al atom)

Figure S1: Plot of formation enthalpy and temperature as a function of doping elements.

Figure S2: Total energy plot as a function of volume by setting constant c/a ratio.

Figure S3: PDOS of pure MgH_2 and Al_MgH_2.

Figure S4: TDOS of pure MgH_2, Ba_MgH_2, Ca_MgH_2 and Sr_MgH_2.

Figure S5: Total density of states (a) pure MgH_2, (b) Al_MgH_2 by considering HSE06.

Figure S6: TDOS of pure MgH_2, Nb_MgH_2, Ni_MgH_2, Sc_MgH_2, Ti_MgH_2 and V_MgH_2.

Figure S7: PDOS plot of d orbital contribution of Sc_MgH_2, Ti_MgH_2, V_MgH_2, Ni_MgH_2 and Nb_MgH_2.

Figure S8: PDOS of Sc_MgH_2, Ti_MgH_2, V_MgH_2, Ni_MgH_2 and Nb_MgH_2.

Figure S9: ELF for neutral doped MgH_2.

Figure S10: ELF for transition metal doped MgH_2.

Figure S11: IRDF for pure and Al doped MgH_2 (110) surface

System	Bulk modulus (GPa)	% of ΔV (at 10 GPa)
MgH_2	52.33	19.11
Ca_MgH_2	50.49	19.81
Sr_MgH_2	51.27	19.51
Ba_MgH_2	48.40	20.66
Al_MgH_2	51.96	19.25
Ga_MgH_2	47.99	20.84
In_MgH_2	49.32	20.28
Sc_MgH_2	52.06	19.21
Ti_MgH_2	53.92	18.55
V_MgH_2	53.49	18.70
Ni_MgH_2	54.21	18.45
Nb_MgH_2	55.32	18.08

Table S1. The bulk modulus value and corresponding volume change in percentage for pure and doped MgH_2 systems at 10 GPa applied pressure.

We calculate the value of total energy (in eV) of Al doped at second and third layer of MgH_2(110) surface (considering three layer system) with reference to the total energy of Al doped at first layer of MgH_2(110) surface (see Table S2). The total energy of Al doped first layer is taken as 0 eV. From Table S2, the Al doping in second and third layer needs 0.58 eV and 0.64 eV compare to the first layer. Overall we can infer that surface doping is easier compare to underneath doping. The similar text and table is now provided in the supporting information.

**Doping layer of MgH_2 (110)**	**Total Energy (eV)**
First layer	0
Second layer	+0.58
Third layer	+0.64

Table S2. The total energy difference for the three layer doping configuration considering Aluminium atom.

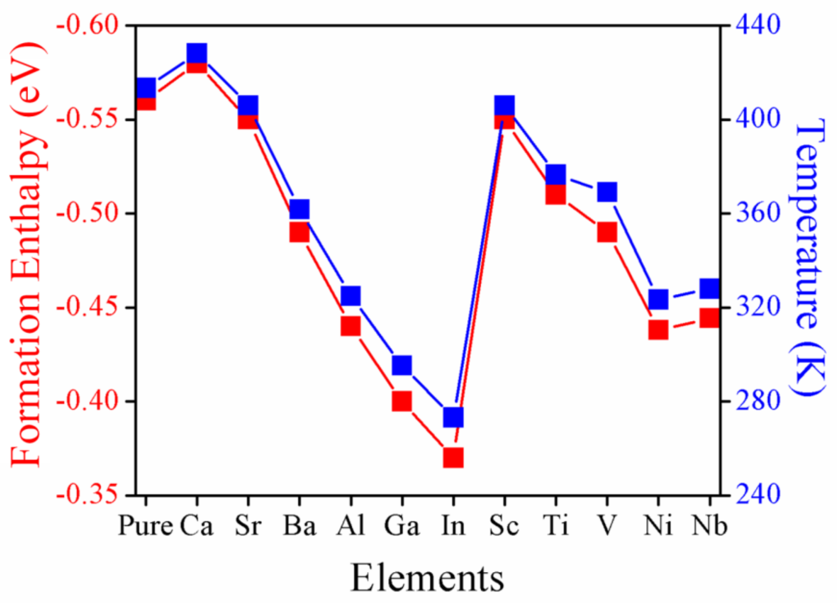

Figure S1. A plot of formation enthalpy (in electron volt) and temperature (in kelvin) as a function of various doping elements. Straight lines with red and blue square symbols denoted the calculated formation enthalpy and desorption temperature for particular elements.

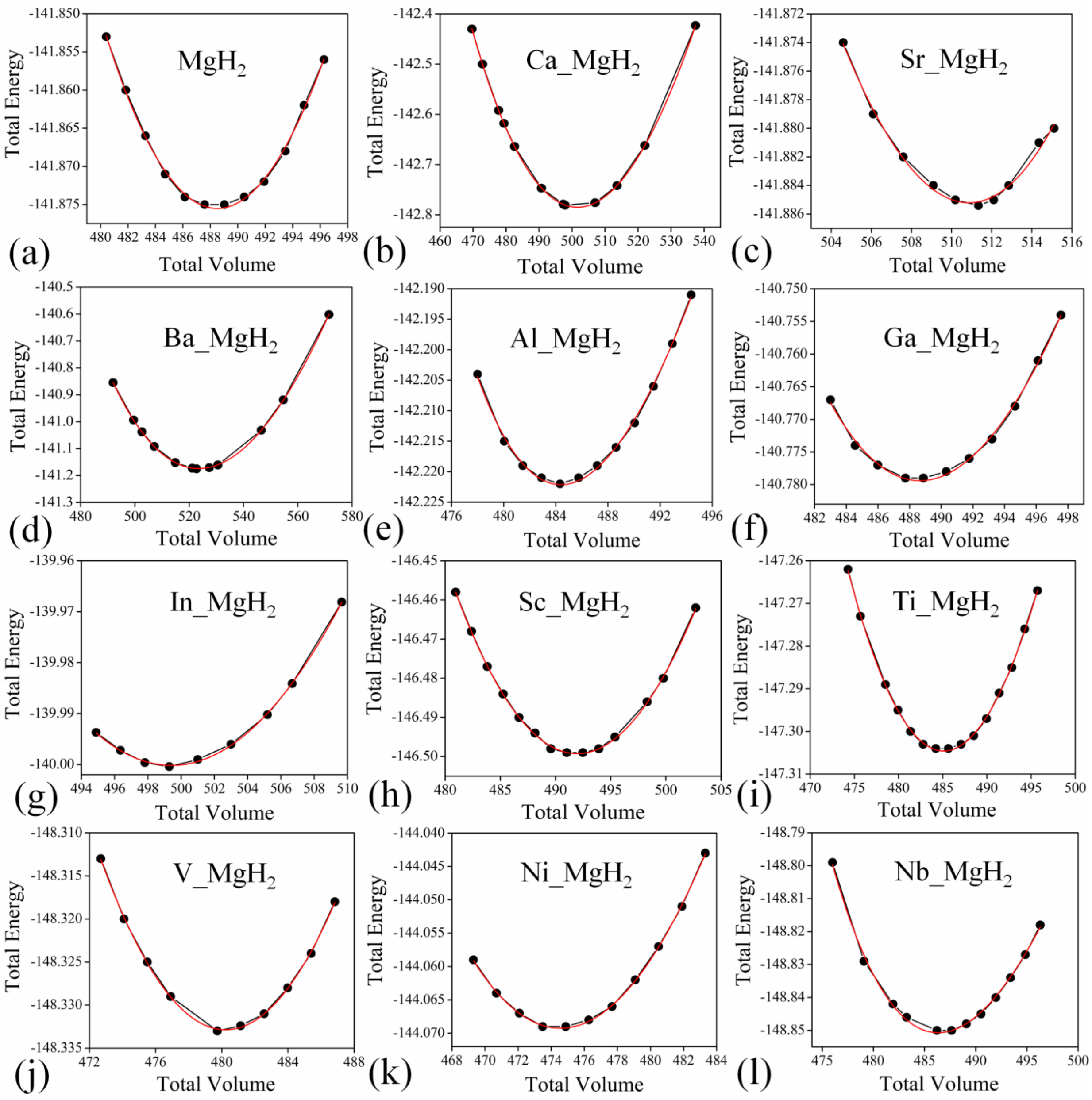

Figure S2. Total energy plot as a function of volume by setting constant c/a ratio of (a) pure and (b-l) all doped MgH_2 systems. The red color lines represent the curve fitted using BM equation.

Bulk Modulus Calculation:

The Birch-Murnaghan's equation of state (E_s) used for finding the bulk modulus of pure and doped MgH_2, and it can be written as

$$E_s = E_0 + \frac{B_0 V}{K(K-1)}\left[K\left(1-\frac{V_0}{V}\right)+\left(\frac{V_0}{V}\right)^K - 1\right] \qquad (1)$$

Where E_0, $B_{0,}$ K, V_0 are the fitting parameters (trail minimum energy, Bulk modulus, first derivative of Bulk modulus and ground state total volume) to make E_s coincide exactly with the parabola curve. The standard error for calculating the bulk modulus value is 0.005.

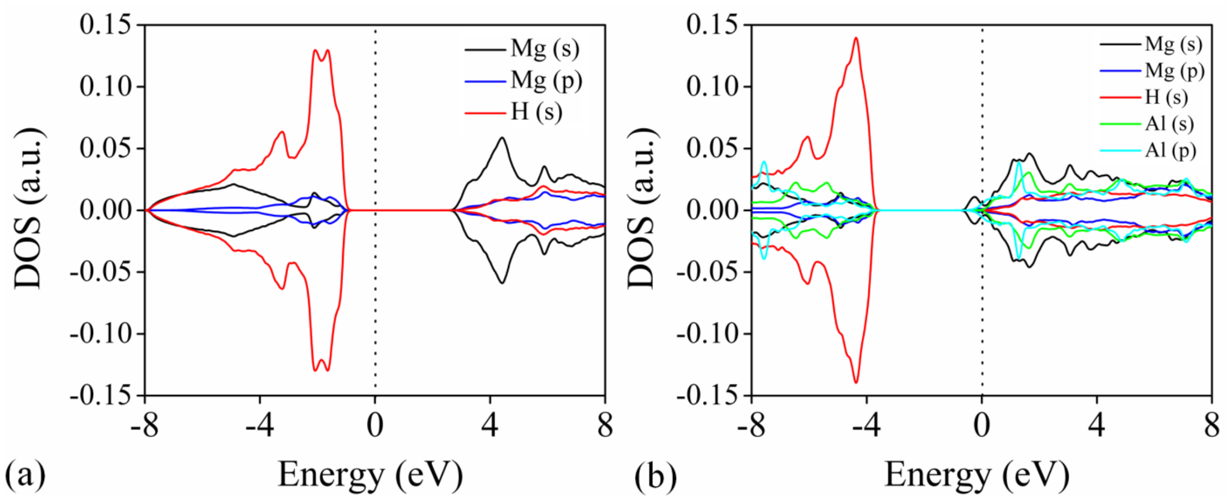

Figure S3. Partial density of states (PDOS) of (a) pure MgH_2 and (b) Al_MgH_2 system. The vertical dotted line denotes the Fermi level.

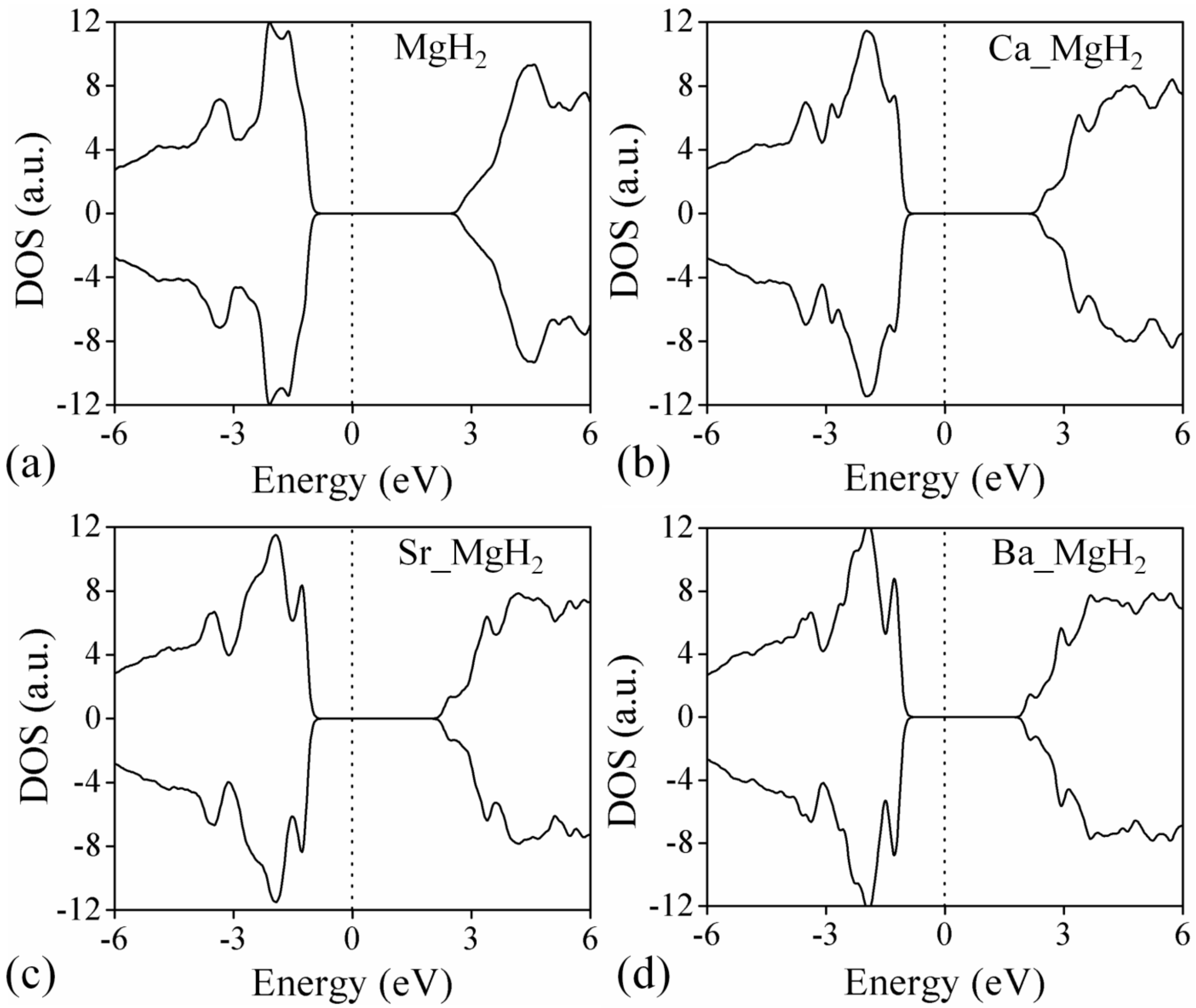

Figure S4. Total density of states (TDOS) of (a) pure MgH_2, (b) Ca_MgH_2, (c) Sr_MgH_2 and (d) Ba_MgH_2. The vertical dotted line denotes the Fermi level.

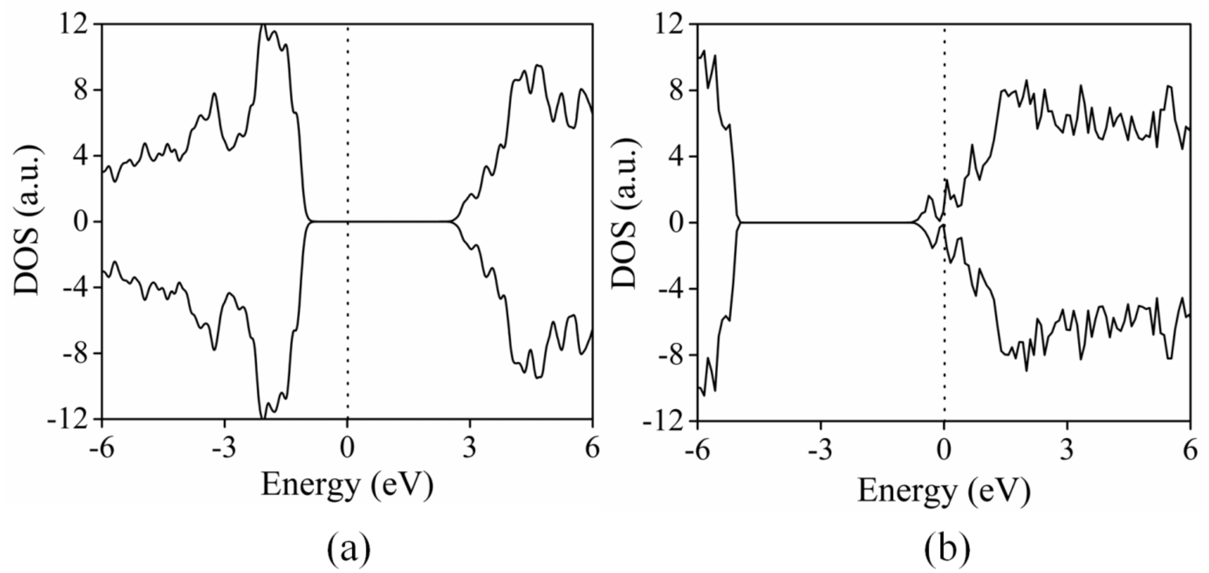

Figure S5. Total density of states (a) pure MgH_2, (b) Al_MgH_2. The vertical dotted line represents the position of the Fermi level.

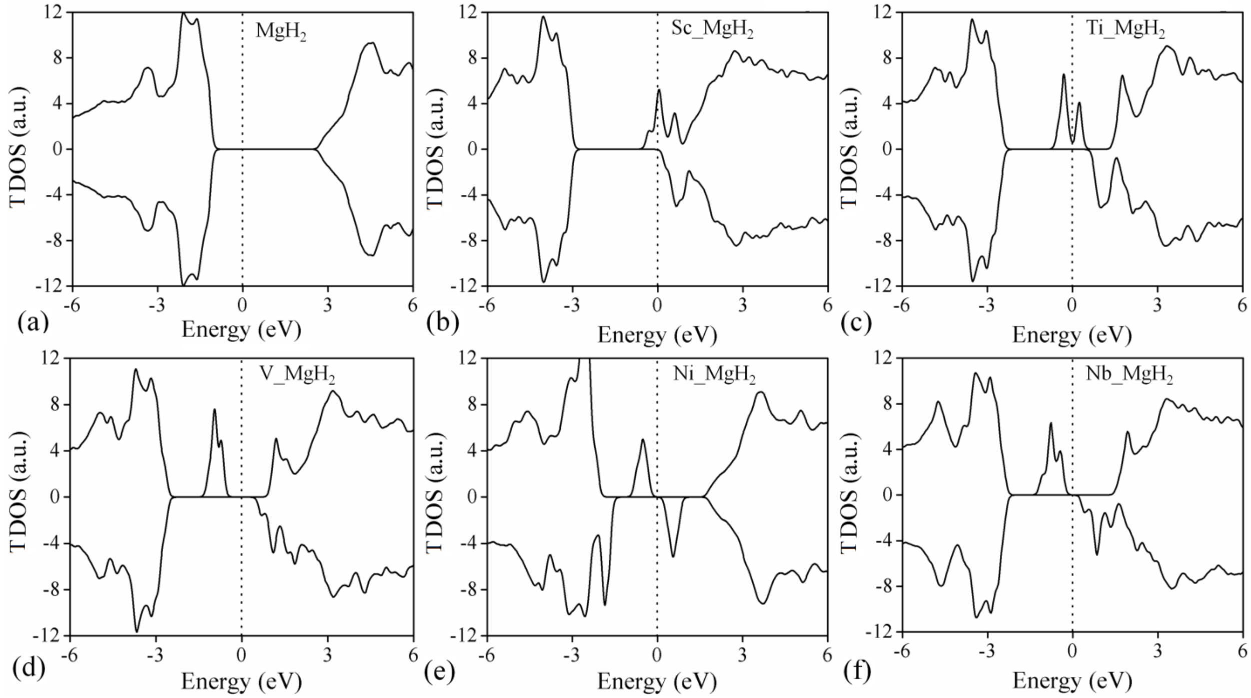

Figure S6. Total density of states (TDOS) (a) pure MgH_2, (b) Sc_MgH_2, (c) Ti_MgH_2, (d) V_MgH_2, (e) Ni_MgH_2 and (d) Nb_MgH_2. The vertical dotted line denotes the Fermi level.

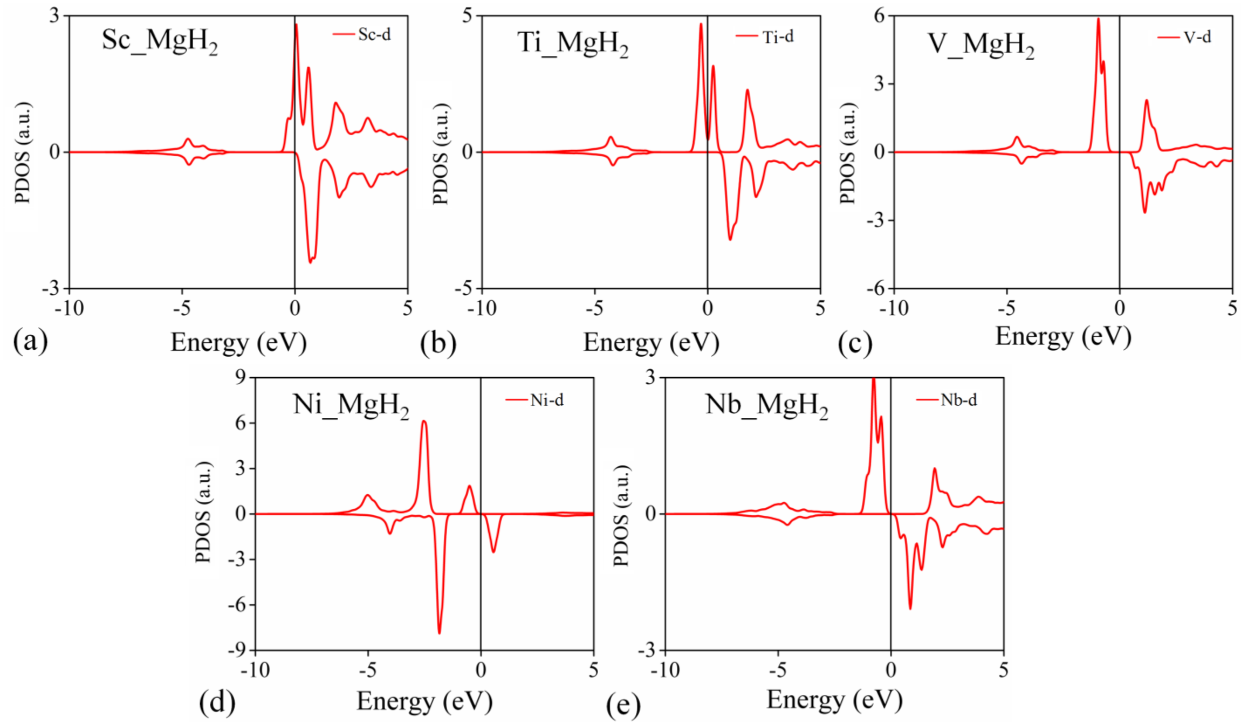

Figure S7. The d orbital projected DOS of (a) Sc_MgH$_2$, (b) Ti_MgH$_2$, (c) V_MgH$_2$, (d) Ni_MgH$_2$ and (e) Nb_MgH$_2$ system. The vertical straight line denotes the Fermi level.

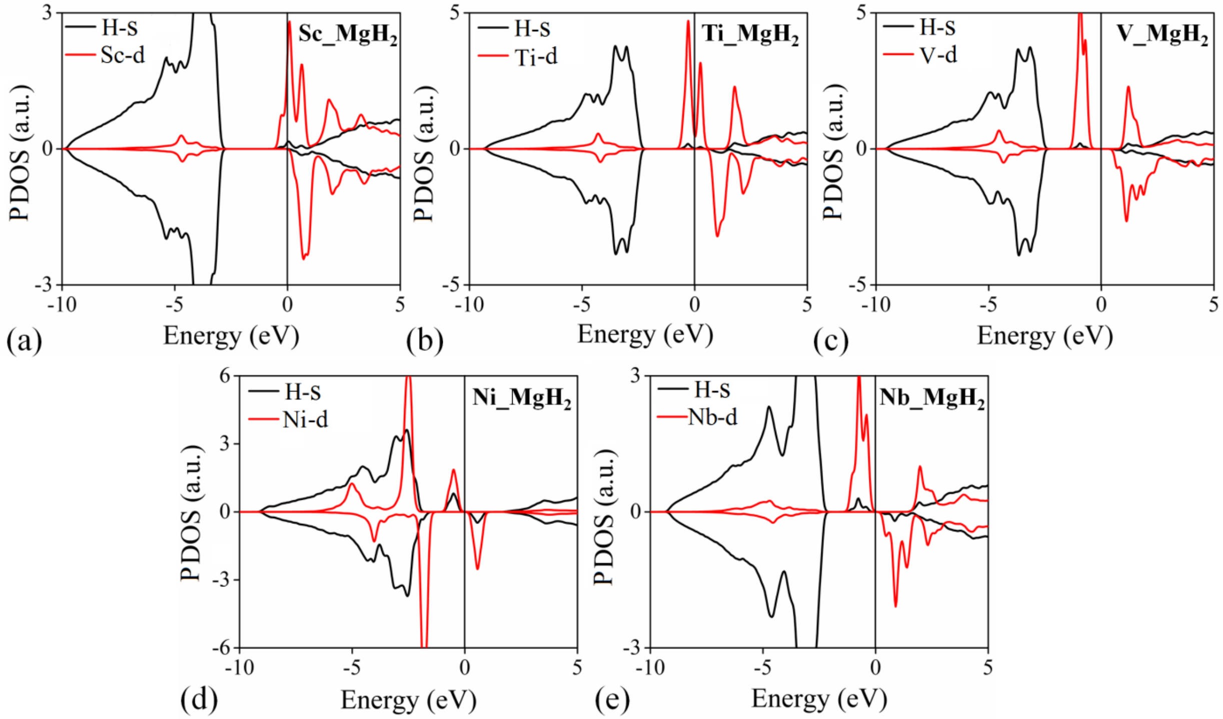

Figure S8. Partial density of states (PDOS) of (a) Sc_MgH_2, (b) Ti_MgH_2, (c) V_MgH_2, (d) Ni_MgH_2 and (e) Nb_MgH_2 systems. The vertical straight line denotes the Fermi level.

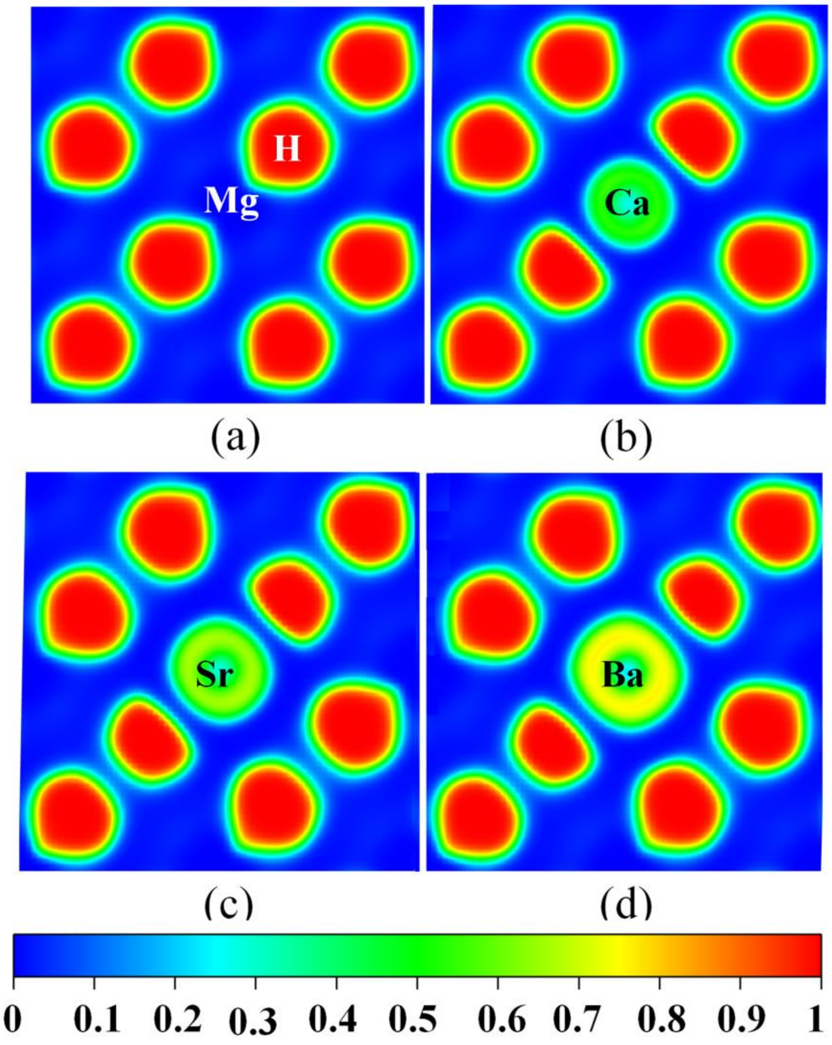

Figure S9. 2D plot of Electron Localization Function (ELF) for (a) pure MgH_2, (b) Ca_MgH_2, (c) Sr_MgH_2 and (d) Ba_MgH_2 systems. Cores are labeled by the respective element. The red and blue region indicates high and low localization respectively, also can be estimate from the bar scale.

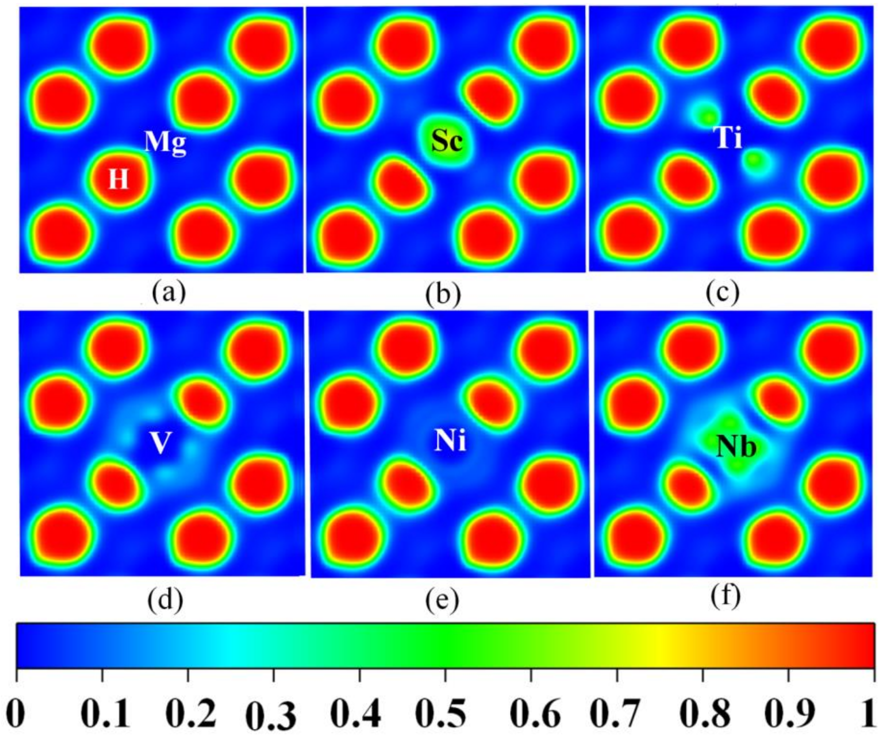

Figure S10. 2D plot of Electron Localization Function for (a) pure MgH_2, (b) Sc_MgH_2, (c) Ti_MgH_2, (d) V_MgH_2, (e) Ni_MgH_2 and (f) Nb_MgH_2 systems. Cores are labeled by the respective element. The red and blue region indicates high and low localization respectively, also can be estimate from the bar scale.

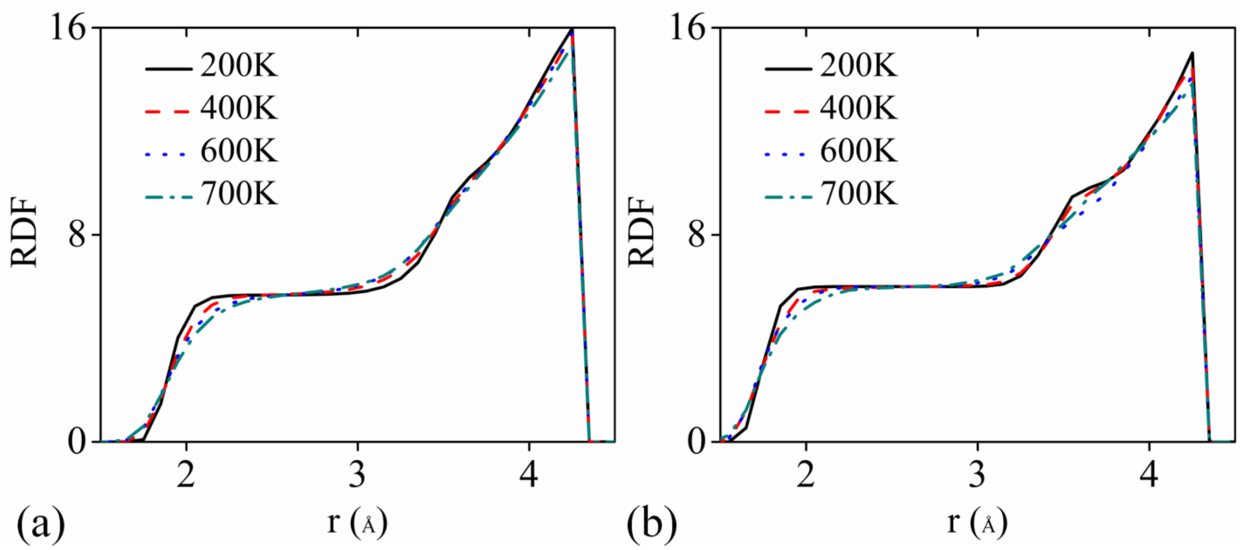

Figure S11. The variation of Integrated Radial distribution function of (a) pure and (b) Al doped MgH_2(110) surface for four different temperatures i.e. 200K, 400K, 600K and 700K.
